# Supplementary material for: The Construction of ceRNA Regulatory Network Unraveled Prognostic Biomarkers and Repositioned Drug Candidates for the Management of Pancreatic Ductal Adenocarcinoma
Source: Curr Issues Mol Biol. 2025 Jun 27;47(7):496. doi: 10.3390/cimb47070496 (PMC12293328; doi:10.3390/cimb47070496)
Supplement: Supplementary file 1 [file cimb-47-00496-s001.zip › Supplementary Table S1.pdf]

Supplementary Table S1. DEcircRNAs in ceRNA network.

**DEcircRNAs**
hsa_circ_0100302
hsa_circ_0102741
hsa_circ_0103211
hsa_circ_0101656
hsa_circ_0104313
hsa_circ_0102751
hsa_circ_0102465
hsa_circ_0100904
hsa_circ_0104168
hsa_circ_0000543
