# Supplementary material for: The Construction of ceRNA Regulatory Network Unraveled Prognostic Biomarkers and Repositioned Drug Candidates for the Management of Pancreatic Ductal Adenocarcinoma
Source: Curr Issues Mol Biol. 2025 Jun 27;47(7):496. doi: 10.3390/cimb47070496 (PMC12293328; doi:10.3390/cimb47070496)
Supplement: Supplementary file 1 [file cimb-47-00496-s001.zip › Supplementary Table S2.pdf]

Supplementary Table S2. DEGs in ceRNA network.

DEGs	
ACSL4	MEF2C
ADAM12	MET
BMPR2	MSN
BTN3A2	MYLK
C1GALT1	P4HB
CCDC80	PGM2L1
CD200	PLSCR1
CD55	PMAIP1
CD86	PSMB9
CDH11	PTGS2
CEP55	QKI
CHST11	RASSF2
CXCL10	RECK
CYP1B1	RND3
DCBLD2	RUNX2
DIO2	SEC23A
EDNRA	SERPINH1
EPHX2	SKIL
ETS1	SLC1A2
ETS2	STC2
FAM72A	STX6
FGD6	TGFBR1
FLNA	THBS2
FN1	TIMP2
FRMD6	TRPS1
GFPT2	UBE2D1
GLI3	VCL
GPX8	WIPF1
HIF1A	ZEB1
ITGA3	ZEB2
KRT7	ZFPM2
LRRFIP1	ZG16
